# Implicit Tasks Reveal Attentional Impairments in Pre‐symptomatic Alzheimer's Disease (AD/ADRD)

**DOI:** 10.1002/alz70857_107384

**Published:** 2025-12-26

**Authors:** Lara C. Krisst, Amir‐Vala Tavakoli, Daw‐An Wu, Shinsuke Shimojo, Astrid M Suchy‐Dicey, Xianghong Arakaki

**Affiliations:** ^1^ California Institute of Technology, Pasadena, CA, USA; ^2^ Huntington Medical Research Institutes, Pasadena, CA, USA

## Abstract

**Background:**

Previous research has shown that participants at high‐risk for Alzheimer's disease, indexed by a ‘pathological’ CSF amyloid/tau ratio, exhibit alterations in attentional processing (Hung et al., 2022). We reasoned that if attentional mechanisms regulated implicit tasks, high risk participants would perform worse in implicit tasks, particularly under high attentional load.

**Method:**

We addressed this question by recording EEG data during a response interference paradigm known as the Simon task. Participants are presented with an arrow and asked to report the direction of the arrow (left or right). In the Simon condition, the direction the arrow points is inconsistent with its placement on the screen. This increases attentional load by introducing a spatial discrepancy (high load). Conversely, in the non‐Simon condition, the arrow direction and placement is consistent (low load). Participants respond faster and more accurately when the stimulus and response features (location) are consistent versus inconsistent, referred to as the *Simon Effect*. In this experiment, prior to the arrow stimulus, participants are also primed with an implicit word in the center of the screen (“left” or ”right”). The implicit word prime is either congruent or incongruent with participants’ direction responses. We hypothesized that the effect of congruency (faster reaction times to primes congruent with responses and vice versa) would diminish in the high load condition of high risk participants.

**Result:**

While preliminary behavioral data show that both high and low risk groups display the Simon effect (*p < .05*), there was no significant effect of prime congruence between groups. When examining the EEG data, we found a significant difference in the mean amplitude of the P300 event related potential (ERP), typically indexing conscious decision making, for the low risk group only in the load manipulation (*p < .05*, Cohen's d = 0.49). We also see a significant effect of prime congruence in the low load condition only for both groups (*p < *.05); an earlier N200 ERP component, indexing attentional processes, showed this same prime congruence effect (*p < .05*).

**Conclusion:**

This differential neural response in high‐risk Alzheimer's participants can be interpreted as an early precursor of cognitive impairment.

